# Editorial: Reading Faces and Bodies: Behavioral and Neural Processes Underlying the Understanding of, and Interaction with, Others

**DOI:** 10.3389/fpsyg.2016.01923

**Published:** 2016-12-08

**Authors:** Paola Ricciardelli, Andrew P. Bayliss, Rossana Actis-Grosso

**Affiliations:** ^1^Department of Psychology, University of Milano-BicoccaMilan, Italy; ^2^Milan Centre for NeuroscienceMilan, Italy; ^3^School of Psychology, University of East AngliaNorwich, UK

**Keywords:** face perception, gaze cueing, body language, atypical development, social information processing

The ability of individuals to understand other people as beings who have intentional and mental states is fundamental to adapt to our social world. To this end, our perceptual and neural systems have evolved to extract useful information from faces and moving bodies of other humans to allow reciprocal social interactions and communication.

A central source of socially meaningful cues is the face and eye gaze, which can be visually analyzed to understand a person's emotions, focus of attention, intentions, beliefs, and desires. All of this body of information, although complex, is easily detected and used by people to go beyond a person's facial appearance to make inferences about personal dispositions and personality traits, such as trustworthiness.

The contributions of this Research Topic have addressed through different methodologies and techniques how we process and integrate the different types of information coming from static and dynamic faces and moving bodies and, on the other hand, how person categorization cues influence the way in which we process faces. The issues emerged from behavioral, neuropsychological, computer, and neurophysiological studies are briefly reviewed along with some remarks on future research directions and outstanding questions.

The specificity and the importance of faces as visual stimuli was addressed in the study by Shyi and Wang, who, by mean of a face composite task, tested the possibility that the top-half of a face might induce stronger holistic processing than the bottom-half counterpart. Their results show instead that holistic processing may distribute homogeneously within an upright face.

The ability of adults in decoding child facial expressions was studied by Gadea et al. These authors analyzed the relation between the facial expressions of a group of children when they told a lie and the accuracy in detecting the lie by a sample of adults, finding that the lies expressed with emotional facial expressions are more easily recognized by adults than the lies expressed with a “poker face.” They also correlated the accuracy of the lie detectors with their subclinical traits of personality disorders. It was found that the presence of an emotion helps the observer to read the mind of the other person and highlight a modulatory effect of personality traits on this ability. Moreover, the interaction between facial cues as an index of emotional internal state and dynamic emotional expressions performed with faces by both an actor and the observer has been investigated by Hyniewska and Sato. With their study they show that the evaluation of an emotional face is influenced not only by the emotional expression of the face to be judged, but also by the emotional expression of the face of the judging person.

Lewinski showed that people are not very accurate at recognizing neutral faces as neutral. By comparing human performance with that of the automated facial coding (AFC) software he found that the computer software was far more accurate than people. This finding opens up new questions on the exact mechanism which can explain this discrepancy and what is the functional meaning and the advantage of seeing a face as emotional.

An important role in face processing can be played by the fact that in everyday life the external (e.g., hair style) and inner components of the face are not seen in isolation. In this respect, the paper by Saegusa et al. showed that attractiveness judgments of hair surrounding a task-irrelevant face were always influenced by the attractiveness of the face itself. This study provides evidence that visual attractiveness information, relevant for person categorization and personality trait inference (Dion et al., 1972), is integrated at the perceptual level. An outstanding issue for future research concerns the temporal dynamic of this integration and where within the human brain (e.g., in the occipitotemporal cortex) it occurs.

However, not only facial cues provide crucial information regarding a person's internal state. In every-day situations, body language or “bodily kinematics” are equally important, especially when facial signals are unavailable to the observer. A growing body of evidence shows that body motion cues are also a core component of social interactions and concur to make the first impression of a person. Actis-Grosso et al. directly compared pictures of static emotional faces with body motion cues (i.e., biological motion display) to test their efficacy in conveying emotions. They found that emotions are not recognized in the same way but some emotions (i.e., sadness) are better recognized when conveyed by static faces whereas others (i.e., fear) by motion displays.

With regard to how face and body motion cues may contribute to social understanding in typical and atypical population, it is becoming apparent that variance in face recognition among the general population is much higher than previously thought. Albonico et al. show that motion improves face recognition performance of poor face recognizers, but does not improve that of those who already find face recognition easy. In their study, Actis-Grosso et al. also compared the performance in the recognition of emotions of young adults with Low or High Autistic Traits, finding that the two groups could rely on different cues for the recognition of emotions.

To date little is known about how facial and body cues interact with each other, and with social (e.g., social identification and group membership) and ecological factors to form a unified representation that can guide our perceptions and responses to other people. Jarick and Kingstone based their study on the hypothesis that a cornerstone of non-verbal communication is the eye contact between individuals and the time that it is held. In their study they show experimentally that the effect of eye contact, which is considered as a form of body language, can be quickly and profoundly altered merely by having participants, who had never met before, play a game in a cooperative or competitive manner. Laskowska used a more ecologically valid test (the Emotional Intelligence Scale—Faces), in which a mixture of basic and complex emotions (or social emotions) were presented, to assess whether the deficit in facial emotion recognition present in Parkinson's (PD) disease is due to impaired sensory processes or impaired decision making ones. They compared PD's patients to healthy controls and to a group of patients with schizophrenia. While in patients with schizophrenia facial emotion recognition seems to originate only from a generalized sensory impairment, PD's patients showed both a decreased sensitivity and a change in response bias compared with healthy controls. This study indicates that when a more ecological approach is taken it provides a better differentiation of the origins underling everyday emotion recognition in pathological populations.

In a similar vein, by using more realistic 3D avatars that suddenly shifted their eyes, thus mimicking more natural social interaction, Dalmaso et al. provide some evidence that in right-hemisphere damaged patients the ability to shift attention in response to eye gaze stimuli (gaze cueing effect) was preserved and that head orientation does not seem to modulate the gaze cueing effect. Therefore, combining the study of neuropsychological patients with that of the processing of social cues provides new hints about both neural and behavioral mechanisms of social attention. In particular, Bobak and Langton cast doubt on the long-held view that gaze cueing does not require top-down control by showing that we do not follow gaze direction when working memory capacity is occupied.

There is not a full theoretical account of how we process, integrate, and interpret the various social signals from a visual image. In an ERP study Del Zotto and Pegna addressed the issue about how the brain process positive and negative facial emotions. In particular, they focused on the interaction between awareness, non-spatial selective attention, and emotion processing. Using a backward masking paradigm, they found that attention and awareness are partially dissociated in emotion processing as indicated by the finding that they affect different EEG components at different processing time.

Finally, Proietti et al. work demonstrates that we look at the faces of people of different ages in different ways. This is important as it adds to data regarding other categories such as ethnicity, using eye tracking as a method to supplement measures such as processing speed to tell us more about processing style and content of in- and out-group individuals. By contrast, the studies by Cañadas et al. and Jacquot et al. respectively provide new evidence on how person categorization and person knowledge can bias cognitive processes. Cañadas et al. show that when learning about the reliability of people in a trust economic game, participants generalize the positive behavior of white faces to other members of that group, while they are sensitive to individual behavior of black faces. On the other hand, Jacquot et al. show that even people that you believe to be incompetent can alter your own metacognitive appraisal of your accuracy at a task. That is, after making a 2AFC judgment, seeing a video of a person nodding their head boosts confidence that one's decision was correct and seeing a head shake reduces this. The effect is smaller but still present even if the person in the video is known to be incompetent. Jacquot et al. also used facial EMG and showed smile-muscle activity only when competent people nodded their head following difficult judgments.

In conclusion, the variety of approaches and methods employed by the studies included in this topic highlights the need to adopt a multidisciplinary perspective to reach a full theoretical account of how we extract, process and interpret the various social signals coming from a person. The new account should integrate information from the face and body as well as social and contextual information, thus helping also to advance current models of face processing. What should still be addressed in future research, for example, is how personality inferences derived from the person's perceptual appearance bias cognitive processes involved in the understanding of others. In future studies, comparing groups of individuals in normal and pathological conditions might help to better understand the interplay between individual differences and social perception. We hope that the papers included here can stimulate and guide research in social cognition and social neuroscience by bringing together research in the field of cognitive and social psychology.

## Author contributions

PR: Planned the topic and edited the majority of papers included in the topic. AB: Edited some papers included in the topic. RA: Planned the topic and edited some papers included in the topic.

## Funding

This work was supported by a grant from the University of Milano-Bicocca (Fondo di Ateneo 2014) to PR and to RA.

### Conflict of interest statement

The authors declare that the research was conducted in the absence of any commercial or financial relationships that could be construed as a potential conflict of interest.
